# Generalized severe myalgia and oedema: a case of fasciitis associated with mixed connective tissue disease

**DOI:** 10.1093/rap/rkac059

**Published:** 2022-07-27

**Authors:** Kentaro Noda, Takashi Shimoyama, Haruyasu Ito, Ken Yoshida, Daitaro Kurosaka

**Affiliations:** Division of Rheumatology, Department of Internal Medicine, The Jikei University School of Medicine, Tokyo, Japan; Division of Rheumatology, Department of Internal Medicine, The Jikei University School of Medicine, Tokyo, Japan; Division of Rheumatology, Department of Internal Medicine, The Jikei University School of Medicine, Tokyo, Japan; Division of Rheumatology, Department of Internal Medicine, The Jikei University School of Medicine, Tokyo, Japan; Division of Rheumatology, Department of Internal Medicine, The Jikei University School of Medicine, Tokyo, Japan

Key message

- Fasciitis might be a cause contributing to myalgia in patients with MCTD.

Dear Editor, MCTD, a concept proposed by Sharp *et al.* [1] in 1972, describes disease complicated with mixed status among SSc, SLE and PM, in addition to common characteristics including the presence of anti-RNP antibodies and RP. It has been reported that myalgia is common in MCTD, even when myositis is not detected histopathologically [2]. However, little is known about the causes of myalgia other than myositis. We describe a case report of MCTD with severe myalgia caused by fasciitis. Informed consent was obtained for the publication of this paper.

A 64-year-old Japanese woman was referred to us and hospitalized in our department because she had difficulty moving her extremities owing to severe myalgia. Five months earlier, she had noticed RP in her fingers. One month earlier, she had developed polyarthralgia, pain in the upper arm and thigh, and oedema in the lower legs. A physical examination revealed bilateral swollen fingers, severe tenderness in her upper legs and upper arms, oedema in her face and bilaterally in her hands, fingers and lower legs. Fine crackles were faintly audible in the bilateral lower lung fields. Her heart sounds were clear, without audible murmurs. We could not evaluate muscle weakness because of severe pain in the extremities. A laboratory examination showed elevated serum levels of creatine kinase and aldolase [330 U/l (normal range <153 U/l) and 21.1 IU/l (normal range <5.9 IU/l), respectively]. ANAs were present at a titre of 1:5120 in a speckled pattern. Anti-Sm and anti-dsDNA antibodies were negative. Anti-U1-RNP antibody was positive at 550 U/ml. A CT scan of the lung revealed a small amount of pleural effusion and very mild reticular opacities. Ultrasonic cardiography showed no evidence of pulmonary hypertension and preserved left ventricular function. A diagnosis of MCTD was made according to criteria proposed by the Ministry of Health and Welfare of Japan [3]. Given that the patient complained of severe myalgia in her thigh and upper arm, we performed muscle MRI to detect the cause. A gadolinium-enhanced fat-suppressed T1-wedged MRI of the thigh showed that the fascia of the gracilis and hamstring muscles was enhanced with high signal intensity (Fig. 1A). Short inversion time recovery image of the bilateral upper arms showed areas of high signal intensity in fascia of the biceps and triceps brachii muscles (Fig. 1B, C). *En bloc* biopsies of skin to the left biceps brachii showed almost intact muscle fibres (Fig. 1E) and mild-to-moderate mononuclear cell infiltration around small vessels in the connective tissue between the deep fascia and muscles (Fig. 1D). No eosinophils were observed in the fascia. Immunohistochemical analyses showed that CD4^+^ cells were mainly present among the inflammatory mononuclear cells around the fascial and intramuscular small blood vessels, whereas CD8^+^, CD68^+^ and CD20^+^ cells were scattered. Given the MRI and histopathological findings, we concluded that fasciitis but not myositis caused severe muscle pain in this case. Thirty milligrams of prednisolone (0.5 mg/kg) was administered to treat fasciitis complicated with MCTD, which immediately improved myalgia and oedema, and the prednisolone was tapered to 10 mg over 2 months.

**Fig. 1:**
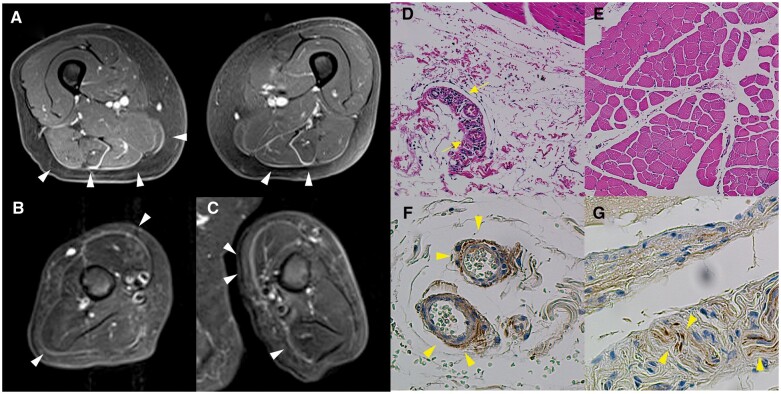
MRI and histopathological findings (**A**) A gadolinium-enhanced fat-suppressed T1-wedged MRI of the thigh shows that the fascia of the gracilis and hamstring muscles is enhanced with high signal intensity (white arrowheads). (**B**, **C**) Short inversion time recovery image of the bilateral upper arms (B: right upper arm; C: left upper arm) shows areas of high signal intensity in the fascia of biceps and triceps brachii muscles (white arrowheads). (**D**) Haematoxylin- and Eosin-stained left triceps branchii muscle tissues show mild-to-moderate mononuclear cell infiltration around small vessels in the connective tissue between the deep fascia and muscles (yellow arrows). (**E**) Muscle fibres were almost intact in the same section as panel D (original magnification ×100 in panels D and E). (**F**) Substance P-immunoreactive fibres (brown; yellow arrowheads) were innervated around small vessels in subcutaneous tissues adjacent to the deep fascia. (**G**) A few substance P-immunoreactive fibres (yellow arrowheads) were present around small arteries in connective tissue between the fasciculus in the same section as panel F (original magnification ×200 in panels F and G).

The most remarkable characteristic in this case is that fasciitis was detected histopathologically instead of myositis, although the patient showed severe myalgia. We reported that myalgia in patients with DM and PM was attributable to fasciitis rather than myositis [4]. Clinically, when performing a muscle biopsy, patients feel pain, especially when cutting into the fascia because, as noted in a rat model, free nerve endings abundantly distributed throughout the fasciae are associated with nociception [5]. However, the muscle fibers of rats are not innervated by substance P-immunoreactive fibres [6]. In this case, substance P-immunoreactive fibres were present around small vessels in subcutaneous tissue adjacent to the deep fascia (Fig. 1F) and around small arteries in connective tissue between the fasciculus (Fig. 1G), but not in muscle fibres. Therefore, we speculated that inflammatory cytokines from mononuclear cells in subfascial tissues sensitized sensory nerve fibres in subcutaneous tissues adjacent to the deep fascia, leading to severe myalgia. The patient had severe oedema in the face and extremities. However, no specific reasons for oedema were detected by laboratory examination and ultrasonic cardiography. Substance P strongly enhances blood vessel permeability. Neurogenic inflammation related to neuropeptides, including substance P secreted from free nerve endings, might be involved in systemic oedema.

Matsuda *et al.* [7] reported a case of fasciitis complicated with MCTD. That case corresponds to our case in terms of the manifestation of severe myalgia. We reported that three of six patients with anti-RNP antibody-associated myositis showed fascial involvement on MRI [8]. Taken together, myalgia related to fasciitis complicated with MCTD might be more frequent than previously thought. Further studies are required to confirm the association between fasciitis and MCTD.

We report a unique case of MCTD with severe myalgia and fasciitis. Fasciitis can lead to severe myalgia. When patients with MCTD show severe myalgia, we should suspect the presence of myositis and fasciitis.

*Funding*: This work was supported by JSPS KAKENHI grant number 22K08551 and Japanese Non-surgical Orthopedics Society （JNOS） grant number JNOS202101.

*Disclosure statement*: The authors have declared no conflicts of interest.

## Data Availability

The data underlying this article will be shared on reasonable request to the corresponding author.
